# Editorial: NETosis, complement activation and pattern recognition at the intersection of inflammatory disorders

**DOI:** 10.3389/fimmu.2025.1594478

**Published:** 2025-03-28

**Authors:** Nibhriti Das, Geeta Rai, Taruna Madan, Uday Kishore

**Affiliations:** ^1^ Department of Biochemistry, All-India Institute of Medical Sciences, New Delhi, India; ^2^ Department of Molecular and Human Genetics, Institute of Science, Banaras Hindu University, Varanasi, India; ^3^ Division of Development Research, Indian Council of Medical Research, New Delhi, India; ^4^ Department of Veterinary Medicine, United Arab Emirates University, Al Ain, United Arab Emirates; ^5^ Zayed Centre for Health Sciences, United Arab Emirates University, Al Ain, United Arab Emirates

**Keywords:** neutrophil, NETs (neutrophil extracellular traps), autoimmunity, cancer, dementia, retinal neuropathy

## Introduction

Cross-talk between the innate immune mechanisms such as NETosis, complement activation and pattern recognition, is integral to an effective host defence and regulation of sterile inflammation. Dysregulated interactive balancing acts among the three mechanisms may lead to an increased susceptibility to infections, autoimmune diseases and cancer. The antimicrobial death mechanism – NETosis (Neutrophil extracellular trap formation) is a key defensive armamentarium of neutrophils to trap invading pathogens. Using diverse pattern recognition receptors including TLRs, Fc receptors, and complement receptors, neutrophils sense the presence of pathogen-associated molecular patterns (PAMPs) and damage-associated molecular patterns (DAMPs) in an inflammatory milieu and release the extracellular traps. DAMP-induced sterile inflammation promotes excessive infiltration of neutrophils to the target tissue as well as potent activation of complement, leading to rapid generation of anaphylaxins, C3a and C5a. Interestingly, the first subcomponent of the complement classical pathway, C1q, as well as Mannan-binding lectin (MBL), the first subcomponent of the complement lectin pathway, also function as pattern recognition receptors. The integrant of neutrophils, majorly the antimicrobial peptides and extracellular DNA, activates the components of the complement system, which in turn, augments the inflammatory process. NETs release more DAMPs, thus, fuelling a vicious cycle of amplified inflammation. In addition to the innate immune defence, the NETing neutrophils also contribute to the pathogenesis of autoimmune diseases and cancer. The biomarkers of NETosis, particularly neutrophil elastase and myeloperoxidase-DNA complex, act as potential autoantigens in various autoinflammatory disorders such as Systemic Lupus Erythematosus (SLE), Rheumatoid Arthritis (RA), diabetes, and psoriasis. NETs', DNA networks are embedded with histones and elastase that are potentially damaging to the host. The pathophysiological role of complement-driven NET release in varied inflammatory disorders is poorly understood. Various cancers, including pancreatic cancer, have shown upregulated NETs. Hence, it is critical to decipher the functional entities and intensive interaction between NETosis, complement components and pattern recognition in regulating the host immune responses.

The objective of this Research Topic compilation was to understand the mechanisms and factors involved in the interplay between the three prominent innate immune processes – NETosis, complement activation and pattern recognition, in inflammatory mechanisms and their significance in the pathophysiology and therapeutic advancements in inflammatory disorders. While the research in this context essentially addresses their translational implications, the goal was to explore a new avenue in the management of inflammatory disorders.

The manuscript by Priya et al. titled “*Dexamethasone and IFN-γ primed mesenchymal stem cells conditioned media immunomodulates aberrant NETosis in SLE via PGE2 and IDO*” offers a promising strategy to manage SLE by targeting NETosis. SLE remains a challenging autoimmune disorder, characterised by a dysregulated immune response, uncontrolled inflammation, and autoimmunity. Central to the pathogenesis of SLE is NETosis, which serves as an immunogenic trigger and exacerbates the disease by releasing autoantigens such as dsDNA and citrullinated proteins. The quest to regulate NETosis has thus emerged as a crucial therapeutic target in the management of SLE. The study shows that conditioned medium from Wharton’s Jelly-derived mesenchymal stem cells (WJ-MSCs), pre-conditioned with dexamethasone (DW) or IFN-γ (IW), reduces excessive NET formation in SLE patients *in vitro* and also in a preclinical lupus model. This reduction in NET formation was accompanied by a marked reduction in reactive oxygen species (ROS) generation, a key mediator of NETosis, indicating a potential mechanism for the observed therapeutic effects. Moreover, the study identifies prostaglandin E2 (PGE2) and indoleamine 2,3-dioxygenase (IDO) as the central immunomodulatory factors responsible for mediating the effects of the conditioned medium. The inhibition of either PGE2 or IDO led to a significant reduction in the suppression of NETosis, further validating their critical role in regulating this pathological process. Interestingly, while the TGF-β pathway was implicated, inhibition studies revealed that TGF-β alone was not as crucial in modulating NETosis as PGE2 and IDO. The therapeutic efficacy of the DW and IW preconditioned medium was further demonstrated in combination with standard SLE therapies, such as hydroxychloroquine (HCQ). The combination treatment showed enhanced suppression of NETosis compared to HCQ alone, suggesting that these novel strategies could augment the effects of existing treatments and potentially reduce the required dosage of traditional drugs, minimizing the side effects often associated with long-term corticosteroids.

Another manuscript titled *“Plasma from patients undergoing allogeneic hematopoietic stem cell transplantation promotes NETosis in vitro and correlates with inflammatory parameters and clinical severity”* elucidates the critical role of NETosis in the pathogenesis of acute graft-versus-host disease (aGVHD) that develops in post-allogeneic hematopoietic stem cell transplantation (allo-HSCT). López-Andrade et al. evaluated the potential of the plasma of allo-HSCT to induce NETosis using plasma samples from 19 allo-HSCT patients and neutrophils isolated from Healthy controls. Using the optimal cut-off value by ROC analysis, patients were classified into two groups: plasma that triggered NETosis (NETs+IC group) and those that did not (NETs-IC group). Most importantly, a significant correlation of the NETs induction capacity (NETsIC) of a study participant with the serum levels of IL-6 and IL-8 cytokines, increased duration of post-transplantation, pro-thrombin time (PT), fibrinogen levels, modified endothelial activation and stress index (M-EASIX) score and occurrence of acute graft-versus-host disease (aGVHD) has endorsed that NETsIC is a critical factor to predict post-allo-HSCT morbidity.

In an extension to the current theme, NETs have been investigated in multiple aspects of tumorigenesis. However, an increased NETosis has never been evidenced in plasma or in the tumour microenvironment of Bladder Cancer (BC) patients. In a well-designed study, Herranz et al. evaluated the increased NETosis in plasma and tumour tissue of BC patients to ascertain whether it is mediated by a reduced DNase I activity and degradation, and to explore novel therapeutic interventions. Plasma samples were obtained before surgery; a formalin-fixed paraffin embedded tumour tissue samples were obtained from 71 patients and plasma samples were collected from 64 age-sex matched controls. NETs markers (cell-free DNA, calprotectin, nucleosomes and neutrophil elastase) and the DNase I activity in plasma with specific assays and NET markers in BC tissues were monitored by immunofluorescence. In addition, the ability of BC and control plasma to degrade *in vitro*-generated NETs were determined and the ability of the approved recombinant human DNase I (rhDNase I, Dornase alfa, Pulmozyme^®^, Roche) to restore the NET- degradation ability of plasma was determined *in vitro*. Apparently, this is the first report demonstrating that the BC patients have an increased NETosis systemically and in the tumour microenvironment. This increase, in part, is caused by an impaired DNase I-mediated NET degradation. Remarkably, this defect could be restored to normal *in vitro* with the approved Dornase alfa; thus, Pulmozyme^®^ could become a potential therapeutic tool to locally reduce BC progression. BC is the 12th most frequent cancer worldwide, with an incidence of more than half a million new cases per year. To date, very few studies have explored the role of NETs in BC, and possible implications in the treatment. This study therefore sheds new light on the understanding of NETosis in BC and manipulation of DNAse I activity as a therapeutic target in the management of the disease.

In a continuation of studies highlighting the significance of NETs in disease mechanisms, Hao et al. examined, via transcriptomics, the expression of NET-associated genes in diabetic retinopathy (DR), where neutrophil infiltration aggravates the inflammatory processes in association with endothelial cells. However, NETs can also contribute to the clearance of ageing/aged blood vessels in a bid to retinal regeneration. The transcriptome of NETs from normal and DR individuals identified 5 key genes in the pathogenesis: CLIC3, GBP2, and P2RY12 appeared to be risk factors for Proliferative DR, whereas HOXA1 and PSAP seemed protective against the development of DR. The identified key genes appear to be involved in oxidative phosphorylation and ribosome functions. The authors also carried out drug predictions targeting P2RY12 that revealed prasugrel, ticagrelor, and ticlopidine as potential therapeutic options.

In a minireview, Aries and Hensely-McBain have eloquently catalogued involvement of neutrophils and its attributes in the animal models of Alzheimer’s disease (AD). AD is the leading cause of dementia globally and its complex underlying immune mechanisms are not fully understood. Not surprisingly, inflammation, and hence neutrophils, have been liked to an exaggeration of dementia in AD. Various murine models of AD (transgenic) have shown neutrophil acquisition in amyloid beta plaques in the brain. Mouse and human studies have revealed an upregulation of neutrophil-associated transcriptomes and a tendency towards NETs release in the AD brains. Thus, controlling neutrophil driven inflammation appears a potential novel therapeutic strategy in AD.

This Research Topic therefore highlights the importance of neutrophils and NETs in a range of pathophysiological mechanisms citing examples of autoimmunity, transplantation, dementia, cancer and diabetic neuropathy. It is worth mentioning that neutrophils have recently been found to be of unique importance in the tumour immune microenvironment (TiME) (including pancreatic ductal adenocarcinoma; PDAC) and their N1 and N2 phenotypes are acquiring an ever so increasing interest in research community. NETs, together with complement components, within the TiME can have a decisive role in tumour prognosis.

